# Supplementary material for: Fully efficient, two-stage analysis of multi-environment trials with directional dominance and multi-trait genomic selection
Source: Theor Appl Genet. 2023 Mar 22;136(4):65. doi: 10.1007/s00122-023-04298-x (PMC10033618; doi:10.1007/s00122-023-04298-x)
Supplement: Supplementary file 1 — Supplementary file1 (PDF 236 KB) [file 122_2023_4298_MOESM1_ESM.pdf]

Supplementary Figures

Fully efficient, two-stage analysis of multi-environment trials with directional dominance and multi-trait genomic selection

Jeffrey B. Endelman
Department of Horticulture, University of Wisconsin-Madison, Madison, WI 53706, USA

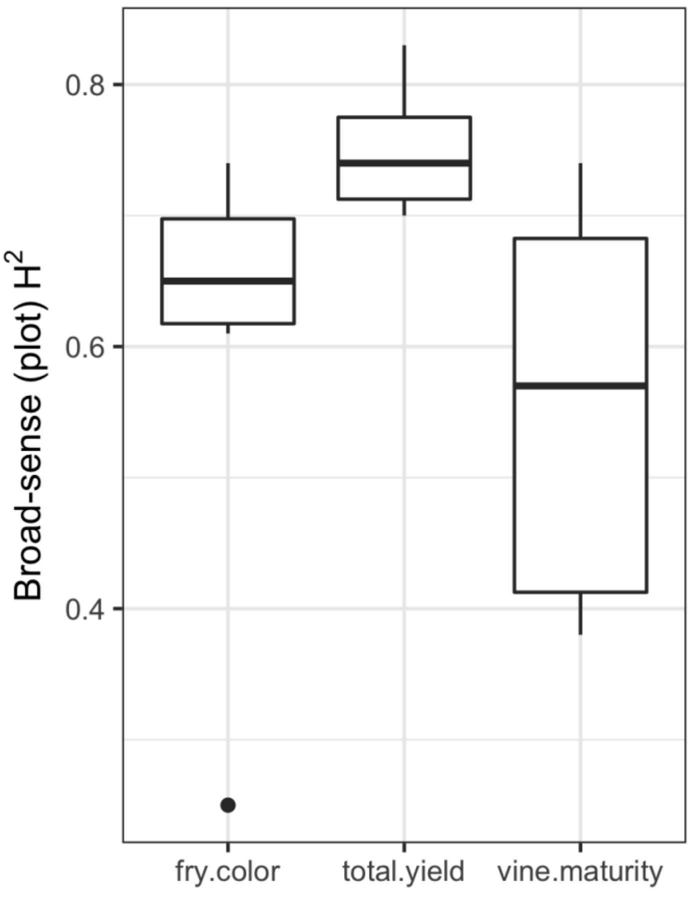

Figure S1. Distribution of the broad-sense heritability (plot basis) by year, from six years of potato yield trials at one location (Hancock, WI).

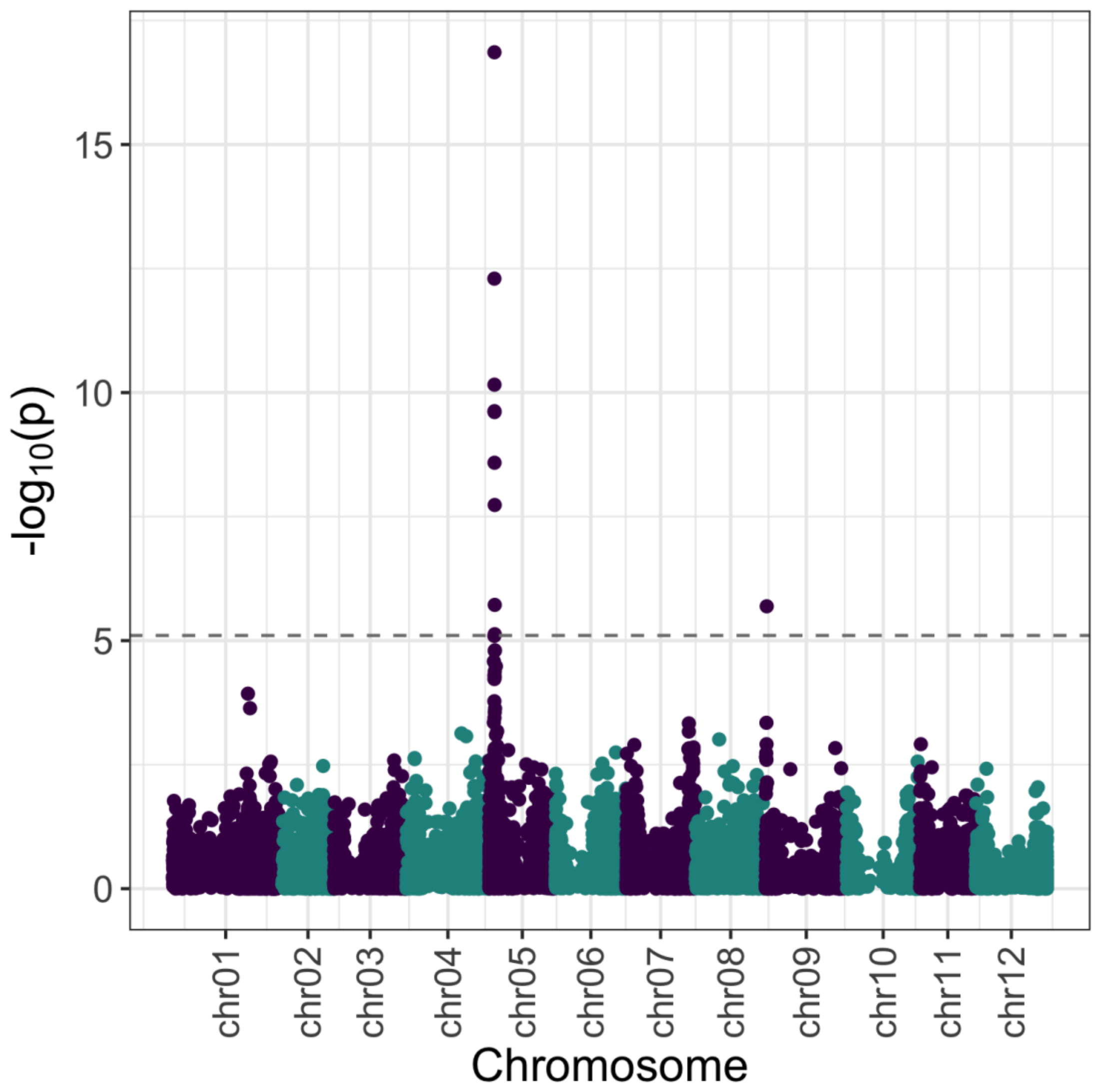

Figure S2. Genome-wide association results for potato vine maturity, using an additive model. The dashed line is the discovery threshold at significance level 0.05, corrected for multiple testing based on the effective number of markers.
