# Supplementary material for: Fully efficient, two-stage analysis of multi-environment trials with directional dominance and multi-trait genomic selection
Source: Theor Appl Genet. 2023 Mar 22;136(4):65. doi: 10.1007/s00122-023-04298-x (PMC10033618; doi:10.1007/s00122-023-04298-x)
Supplement: Supplementary file 2 — Supplementary file2 (PDF 157 KB) [file 122_2023_4298_MOESM2_ESM.pdf]

Supplementary File: Restricted Selection Index

Jeffrey B. Endelman

StageWise uses a new approach to compute the restricted selection index coefficients based on convex optimization software, which allows for inequality and equality constraints on the response. Equality constraints have been historically used for mathematical convenience, even if an inequality better represents the situation. This applies when a response in only one direction (e.g., positive) is acceptable even though no economic weight is given to the trait.

With a single restricted trait, the optimum solution corresponds to zero response, so the inequality constraint provides no advantage. However, with two or more restricted traits, a greater response for the target traits can be observed when using inequality constraints, depending on the genetic correlations.

The `gain` function in StageWise can be used to illustrate this phenomenon. This command was introduced in Vignette 3, in which case the first argument was the output from the `blup_prep` command. The `gain` function computes the matrix of the quadratic form from this data (B^{-1} in Eq. 25, rescaled by the genetic standard deviations), but it is also possible to directly supply this matrix. For simplicity, we will consider selection on true values without dominance, in which case the matrix is the inverse of the additive correlation.

Consider selection on one focal trait, which is correlated with two other traits (values of 0.3 and 0.6) for which a positive response is undesirable. The correlation between the restricted traits will be varied from 0 to 0.9, and the response for the target trait will be computed for two different restricted indices: inequality vs. equality constraints.

```
library(StageWise)
library(ggplot2)
x <- seq(0,0.9,by=0.05)
nx <- length(x)
traits <- paste0("t",1:3)
ineq.response <- eq.response <- NULL

for (i in 1:nx) {
  B <- rbind(c(1,0.3,0.6),c(0.3,1,x[i]),c(0.6,x[i],1))
  dimnames(B) <- list(traits,traits)
  Binv <- solve(B)
  eq.index <- gain(Binv, coeff=c(t1=1, t2=0, t3=0),
       restricted=data.frame(trait=c("t2","t3"),sign=c("=","=")))
  ineq.index <- gain(Binv, coeff=c(t1=1, t2=0, t3=0),
       restricted=data.frame(trait=c("t2","t3"),sign=c("<","<")))
  eq.response <- rbind(eq.response,
                       data.frame(x=x[i],eq.index$table))
  ineq.response <- rbind(ineq.response,
                         data.frame(x=x[i],ineq.index$table))
}

data <- rbind(data.frame(constraints="eq",eq.response),
              data.frame(constraints="ineq",ineq.response))
ggplot(data,aes(x=x,y=response,linetype=constraints,colour=trait)) +
  geom_line() + xlab("t2-t3 correlation") + theme_bw()
```

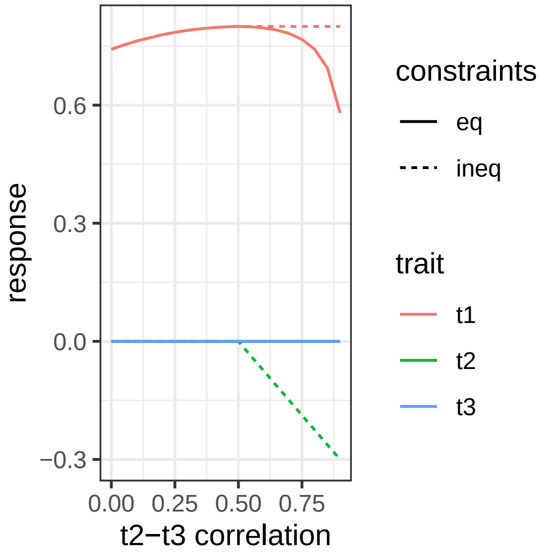

The figure shows that once the t2-t3 correlation exceeds 0.5, higher gains in t1 are possible by allowing a negative response for t2, which is the trait with the lower correlation with t1.
