# Supplementary material for: Fully efficient, two-stage analysis of multi-environment trials with directional dominance and multi-trait genomic selection
Source: Theor Appl Genet. 2023 Mar 22;136(4):65. doi: 10.1007/s00122-023-04298-x (PMC10033618; doi:10.1007/s00122-023-04298-x)
Supplement: Supplementary file 3 — Supplementary file3 (ZIP 14439 KB) [file 122_2023_4298_MOESM3_ESM.zip › StageWise/Vignette2.html]

Vignette 2: Single trait analysis at correlated locations


# Vignette 2: Single trait analysis at correlated locations

This vignette builds on the information in Vignette
1, which covered analysis at a single location. This vignette
illustrates how to model the covariance between multiple locations,
using a dataset of potato yield trials across 8 locations (TX was
excluded) and 6 years, with a population of 336 genotypes (Schmitz Carley et
al. 2019).

```
library(StageWise)
geno.file <- system.file("vignette_data", "geno2.csv", package = "StageWise")
geno <- read_geno(filename=geno.file,ploidy=4,map=FALSE,dominance=TRUE)
```

```
## Minor allele threshold = 5 genotypes
## Number of markers = 5262
## Number of genotypes = 336
```

```
pheno.file <- system.file("vignette_data", "pheno2.csv", package = "StageWise")
pheno <- read.csv(pheno.file)
head(pheno)
```

```
##           id loc     env Yield.Mg.ha
## 1  A01143-3C  CA CA_2011       90.98
## 2  A01143-3C  CA CA_2011       95.12
## 3 AC00206-2W  CA CA_2011       74.44
## 4 AC01151-5W  CA CA_2011       74.44
## 5 AC01151-5W  CA CA_2011       95.12
## 6 AC03452-2W  CA CA_2011       86.85
```

```
table(pheno$loc)
```

```
## 
##   CA   FL   MI   MO   NC   NY   OR   WI 
##  831  841  844  504 1232  869  629  777
```

The input file contains not only a column named ‘env’ for environment
but also one named ‘loc’ for location. The location information is not
explicitly used by `Stage1` but is automatically detected and
retained in the output for use in `Stage2`.

```
ans1 <- Stage1(filename=pheno.file, traits='Yield.Mg.ha')
```

```
## Online License checked out Mon Sep 19 21:40:21 2022
## Online License checked out Mon Sep 19 21:40:22 2022
```

```
head(ans1$blues)
```

```
##       env          id    BLUE loc
## 1 CA_2011   A01143-3C  93.050  CA
## 2 CA_2011  AC00206-2W  74.440  CA
## 3 CA_2011  AC01151-5W  84.780  CA
## 4 CA_2011  AC03452-2W  86.850  CA
## 5 CA_2011  AC05153-1W  49.630  CA
## 6 CA_2011 Accumulator 105.455  CA
```

```
library(ggplot2)
ggplot(data=ans1$fit,aes(x=loc,y=H2)) + stat_boxplot(outlier.color="red") + xlab("Location") + ylab(expression(paste("Broad-sense ",H^2," (plot basis)")))
```

The above figure shows the variation in broad-sense heritability
across locations and years.

When the data frame of BLUEs passed to `Stage2` has a
column labeled ‘loc’, genotype x location effects are included using a
separable covariance structure. The genetic covariance between locations for the highest order genetic effect (i.e., additive when marker data are included) follows a 2nd order factor-analytic (FA2) model. (For non-additive genetic effects, the correlation is constrained at 1.) For large datasets
with many locations (such as this one), I recommend first analyzing it
without marker data, as the computation proceeds more quickly.

Here is a joint analysis of the 8 locations without using the marker
data.

```
ans2a <- Stage2(data=ans1$blues, vcov=ans1$vcov)
```

```
summary(ans2a$vars)
```

```
## $var
##              Variance   PVE
## env               286    NA
## genotype           53 0.360
## g x loc            15 0.100
## g x env            27 0.183
## Stage1.error       53 0.358
## 
## $cor
##       CA    FL    MI    MO    NC    NY    OR    WI
## CA 1.000 0.635 0.691 0.736 0.837 0.686 0.870 0.752
## FL 0.635 1.000 0.670 0.716 0.696 0.702 0.734 0.720
## MI 0.691 0.670 1.000 0.935 0.860 0.930 0.912 0.934
## MO 0.736 0.716 0.935 1.000 0.919 0.996 0.974 1.000
## NC 0.837 0.696 0.860 0.919 1.000 0.899 0.949 0.925
## NY 0.686 0.702 0.930 0.996 0.899 1.000 0.955 0.994
## OR 0.870 0.734 0.912 0.974 0.949 0.955 1.000 0.980
## WI 0.752 0.720 0.934 1.000 0.925 0.994 0.980 1.000
```

As in the single location analysis, running the `summary`
command on the ‘vars’ output shows the partitioning of variance, but now
a g x loc effect is also present. Because g x year effects are not
repeatable and often small, they are not included in the
`Stage2` model.

The FA2 model should have enough complexity for a set of correlated
locations used by a single breeding program, but it may be inadequate
when analyzing disparate locations. The factor loadings returned by
`Stage2` can be used with the function `uniplot`
to visualize model sufficiency and correlation structure (Cullis et al. 2010).

```
uniplot(ans2a$loadings)
```

The squared radius for each location is the proportion of variance
explained (PVE) by the latent factors. With the exception of FL, the FA2
model appears to provide a good representation of the covariance
structure. The numeric PVE values can be obtained as follows:

```
apply(ans2a$loadings,1,norm,type="f")^2
```

```
##        CA        FL        MI        MO        NC        NY        OR        WI 
## 1.0000000 0.5383818 0.8734528 1.0000000 0.9005699 0.9963446 1.0000000 1.0000000
```

The cosine of the angle between locations equals the correlation due
to the latent factors (recall cos(0) = 1). Focusing on the 6 highly
correlated locations-WI,MI,NC,OR,NY,MO-we now add the marker data to
`Stage2` to estimate the additive correlation and predict
breeding values.

```
locs <- c("WI","MI","OR","NY","NC","MO")
blues <- ans1$blues[ans1$blues$loc %in% locs,]
tmp <- sapply(strsplit(names(ans1$vcov),split="_"),"[[",1)
vcov <- ans1$vcov[tmp %in% locs]
```

```
ans2b <- Stage2(data=blues,vcov=vcov,geno=geno,non.add="g.resid")
ans2c <- Stage2(data=blues,vcov=vcov,geno=geno,non.add="dom")
```

```
data.frame(non.add=c("g.resid","dom"), AIC=c(ans2b$aic,ans2c$aic))
```

```
##   non.add      AIC
## 1 g.resid 16715.49
## 2     dom 16685.18
```

The dominance model is selected over the genetic residual model
because of its lower AIC.

```
summary(ans2c$vars)
```

```
## $var
##              Variance   PVE
## env               310    NA
## additive           35 0.211
## add x loc           8 0.048
## dominance          36 0.215
## heterosis           4 0.024
## g x env            24 0.145
## Stage1.error       60 0.357
## 
## $cor
##       MI    MO    NC    NY    OR    WI
## MI 1.000 0.892 0.708 0.883 0.898 0.908
## MO 0.892 1.000 0.897 0.930 0.981 0.941
## NC 0.708 0.897 1.000 0.719 0.831 0.697
## NY 0.883 0.930 0.719 1.000 0.941 0.961
## OR 0.898 0.981 0.831 0.941 1.000 0.959
## WI 0.908 0.941 0.697 0.961 0.959 1.000
```

```
uniplot(ans2c$loadings)
```

The correlation matrix and uniplot, which are based on the additive
values, show that yield in NC was somewhat different compared to the
other five sites.

Compared to the single location analysis in Vignette 1, an additional
argument is needed for the `blup` function to specify the
index coefficients, which are interpreted as the relative weight for
each location after standardization to unit variance. The following code
compares the reliability for BV predictions in WI vs. NC.

```
prep1 <- blup_prep(data=blues,vcov=vcov,geno=geno,vars=ans2c$vars)

WI.index <- c(WI=1, NC=0, OR=0, NY=0, MO=0, MI=0)  
NC.index <- c(WI=0, NC=1, OR=0, NY=0, MO=0, MI=0)  

WI.pred <- blup(data=prep1,geno=geno,index.coeff=WI.index,what="BV")
NC.pred <- blup(data=prep1,geno=geno,index.coeff=NC.index,what="BV")

pred <- merge(NC.pred,WI.pred,by="id")
colnames(pred) <- c("id","BV.NC","r2.NC","BV.WI","r2.WI")

ggplot(pred,aes(x=r2.NC,y=r2.WI)) + geom_point() + coord_fixed(ratio=1) + xlim(0.3,0.8) + ylim(0.3,0.8) + geom_line(data=data.frame(x=c(0.3,0.8),y=c(0.3,0.8)),mapping=aes(x=x,y=y),linetype=2) + theme_bw() + 
  xlab("NC") + ylab("WI") + ggtitle("BV Reliability")
```

The above figure shows that breeding values were predicted with
higher reliability in WI than NC, which is to be expected from the high
correlation between WI and the other four sites (OR,MO,MI,NY) compared
to NC.

The `mask` argument for `blup_prep` can be used
to mask individuals at one or more locations, to explore the accuracy of
prediction into new environments. Whereas the previous analysis
determined the reliability of GEBVs in WI when WI phenotypes are
available, the next analysis excludes WI phenotypes:

```
WI.env <- unique(blues$env[blues$loc=="WI"])

prep2 <- blup_prep(data=blues,vcov=vcov,geno=geno,vars=ans2c$vars,
                   mask=data.frame(env=WI.env))

pred2 <- blup(data=prep2,geno=geno,index.coeff=WI.index,what="BV")

plot.data <- merge(pred2[,c("id","r2")],WI.pred[,c("id","r2")],by="id")
colnames(plot.data) <- c("id","without.WI.pheno","with.WI.pheno")

ggplot(plot.data,aes(x=without.WI.pheno,y=with.WI.pheno)) + geom_point() + coord_fixed(ratio=1) + xlim(0.3,0.8) + ylim(0.3,0.8) + geom_line(data=data.frame(x=c(0.3,0.8),y=c(0.3,0.8)),mapping=aes(x=x,y=y),linetype=2) + theme_bw() + 
  xlab("Without WI phenotypes") + ylab("With WI phenotypes") + ggtitle("WI BV Reliability")
```

As expected, the reliability was higher with WI phenotypes.
