# Supplementary material for: Fully efficient, two-stage analysis of multi-environment trials with directional dominance and multi-trait genomic selection
Source: Theor Appl Genet. 2023 Mar 22;136(4):65. doi: 10.1007/s00122-023-04298-x (PMC10033618; doi:10.1007/s00122-023-04298-x)
Supplement: Supplementary file 3 — Supplementary file3 (ZIP 14439 KB) [file 122_2023_4298_MOESM3_ESM.zip › StageWise/Vignette3.html]

Vignette 3: Multi-trait analysis


# Vignette 3: Multi-trait analysis

In Vignette
1, three traits were analyzed independently in a dataset of 943
potato clones: yield, maturity, and fry color. Like most crops, a large
number of traits are evaluated in potato breeding; in fact, too many to
analyze jointly in StageWise. To make the analysis manageable, I
recommend identifying groups of highly correlated traits based on the
Stage 1 BLUEs. A simple loop can be used for this:

```
library(StageWise)
pheno.file <- system.file("vignette_data", "pheno1a.csv", package = "StageWise")
effects <- data.frame(name=c("block","stand.count"),
                      fixed=c(FALSE,TRUE),
                      factor=c(TRUE,FALSE))
traits <- c("total.yield","vine.maturity","fry.color")
n.trait <- length(traits)

stage1.blues <- NULL
for (i in 1:n.trait) {
  tmp <- Stage1(filename=pheno.file,traits=traits[i],effects=effects)
  if (!is.null(stage1.blues)) {
    stage1.blues <- merge(stage1.blues,tmp$blue,by=c("id","env"))
  } else {
    stage1.blues <- tmp$blue
  }
}
```

```
## Online License checked out Tue Sep 20 17:22:11 2022
## Online License checked out Tue Sep 20 17:22:12 2022
```

```
colnames(stage1.blues) <- c("id","env",traits)
head(stage1.blues)
```

```
##           id       env total.yield vine.maturity fry.color
## 1  A00188-3C Hancock15    66.81187      7.730410  48.28063
## 2  A01143-3C Hancock15   104.41187      8.230410  50.88063
## 3  A09037-6C Hancock15    76.55071      4.923612  54.55500
## 4  A11516-1C Hancock18    45.32170      3.003406  51.51364
## 5  A11520-1C Hancock18    61.24219      3.003407  51.15342
## 6 AAF07847-2 Hancock15    83.85071      7.923612  49.65500
```

```
#Calculate corr matrix for each env
lapply(X = split(stage1.blues,stage1.blues$env),
       FUN = function(data){round(cor(data[,2+1:n.trait]),2)})
```

```
## $Hancock15
##               total.yield vine.maturity fry.color
## total.yield          1.00          0.33     -0.03
## vine.maturity        0.33          1.00      0.03
## fry.color           -0.03          0.03      1.00
## 
## $Hancock16
##               total.yield vine.maturity fry.color
## total.yield          1.00          0.40      0.07
## vine.maturity        0.40          1.00      0.04
## fry.color            0.07          0.04      1.00
## 
## $Hancock17
##               total.yield vine.maturity fry.color
## total.yield          1.00          0.45     -0.06
## vine.maturity        0.45          1.00      0.01
## fry.color           -0.06          0.01      1.00
## 
## $Hancock18
##               total.yield vine.maturity fry.color
## total.yield          1.00          0.32      0.11
## vine.maturity        0.32          1.00      0.03
## fry.color            0.11          0.03      1.00
## 
## $Hancock19
##               total.yield vine.maturity fry.color
## total.yield          1.00          0.19     -0.18
## vine.maturity        0.19          1.00      0.05
## fry.color           -0.18          0.05      1.00
```

The output shows that in four of the five years, the phenotypic
correlation between yield and vine maturity exceeded 0.3, while the
correlation between fry color and these traits was consistently less
than 0.2 in magnitude. We will therefore proceed with a correlated trait
analysis for yield and maturity and combine the results with fry color
later.

The syntax for analyzing multiple traits closely follows the workflow
for one trait:

```
ans1 <- Stage1(filename=pheno.file,traits=c("total.yield","vine.maturity"),
              effects=effects)
names(ans1)
```

```
## [1] "blues" "vcov"  "fit"   "resid"
```

As with the single trait analysis, `Stage1` returns a data
frame of BLUEs and a list of their var-cov matrices. Instead of residual
diagnostic plots, however, the residual covariance matrices are returned
in “resid”. Here is the code to analyze Stage 2 with directional
dominance:

```
geno.file <- system.file("vignette_data", "geno1.csv", package = "StageWise")
geno <- read_geno(geno.file,ploidy=4,map=TRUE,dominance = TRUE)
```

```
## Minor allele threshold = 5 genotypes
## Number of markers = 12242
## Number of genotypes = 943
```

```
ans2 <- Stage2(data=ans1$blue, vcov=ans1$vcov, geno=geno, non.add="dom",
               silent=FALSE)
```

```
## Model fitted using the sigma parameterization.
## ASReml 4.1.0 Tue Sep 20 17:22:53 2022
##           LogLik        Sigma2     DF     wall    cpu
##  1     -3387.277           1.0   1794 17:24:01   66.9 (1 restrained)
##  2     -3352.001           1.0   1794 17:24:57   56.7
##  3     -3328.758           1.0   1794 17:25:56   58.5
##  4     -3322.506           1.0   1794 17:26:54   57.9
##  5     -3321.124           1.0   1794 17:27:52   57.4
##  6     -3321.011           1.0   1794 17:28:49   57.5
##  7     -3321.003           1.0   1794 17:29:47   57.5
##  8     -3321.003           1.0   1794 17:30:45   58.0
```

```
summary(ans2$vars)
```

```
## $var
##              total.yield vine.maturity
## env                 67.7         0.495
## additive            41.4         0.999
## dominance           26.5         0.201
## heterosis            3.9         0.007
## g x env             43.0         0.461
## Stage1.error        31.9         0.998
## 
## $PVE
##              total.yield vine.maturity
## additive           0.282         0.375
## dominance          0.181         0.075
## heterosis          0.026         0.002
## g x env            0.293         0.173
## Stage1.error       0.218         0.375
## 
## $cor.mat
##               total.yield vine.maturity
## total.yield         1.000         0.522
## vine.maturity       0.522         1.000
```

The `summary` command shows the variances and proportion
of variation explained (PVE) as separate tables, as well as the additive
genetic correlation between the traits, which was 0.52. The results show
dominance was more important for yield than maturity.

The next commands in the workflow are `blup_prep` and
`blup`. For the latter, we need to specify the index
coefficients for the standardized traits, which represent their relative
economic weights. For selection on yield without considering maturity,
the code looks like this:

```
prep1 <- blup_prep(ans1$blues, vcov=ans1$vcov, geno=geno,vars=ans2$vars)

index1 <- c(total.yield=1, vine.maturity=0)
GEBV1 <- blup(prep1, geno, what="BV", index.coeff=index1)
```

However, because yield and late maturity are correlated, the above
index will lead to later maturity, which is undesirable. The
`gain` command can be used to compute the expected response
for different indices, in units of intensity x standard deviation (\(i\sigma\)), and its input is the output
from `blup_prep`:

```
gain1 <- gain(input=prep1, traits=c("total.yield","vine.maturity"), 
              coeff=index1)
kable(gain1$table)
```

| trait | response | coeff |
| --- | --- | --- |
| total.yield | 0.686 | 1 |
| vine.maturity | 0.414 | 0 |

```
gain1$plot
```

The above plot is the ellipse of possible responses for the two
traits (in units of \(i\sigma\)). The
dashed red line represents the direction of the index vector, which we
specified to have zero weight for maturity. The blue line segment shows
the response that maximizes genetic merit, which in this case is the
point on the ellipse with the largest value for yield. However, as the
table and figure show, this leads maturity to increase by 0.41\(i\sigma\).

To select for higher yield without increasing maturity, we need a
*restricted* index. Intuitively, we know this requires putting
negative weight on maturity in the index. The optimal index coefficient
can be determined using `gain` by including a table of
constraints with two columns: “trait” and “sign”. The “sign” column can
have one of three symbols for each trait: “=”, “<”, “>”, which
indicate whether the response is \(=
0\), \(\leq 0\), or \(\geq 0\), respectively. In this case, we
want the maturity response to be less than or equal to zero:

```
gain2 <- gain(input=prep1, traits=c("total.yield","vine.maturity"),
              coeff=index1,
              restricted=data.frame(trait="vine.maturity", sign="<"))
kable(gain2$table)
```

| trait | response | coeff |
| --- | --- | --- |
| total.yield | 0.556 | 0.870 |
| vine.maturity | 0.000 | -0.494 |

```
gain2$plot
```

The table output shows the index coefficients to achieve zero
response for maturity while maximizing yield. Comparing with the earlier
table, the yield response decreases from \(0.69i\sigma\) to \(0.56i\sigma\) with the restricted index.
The ellipse plot shows the changes visually.

We can use this information to calculate breeding values for the
restricted index:

```
index2 <- gain2$table$coeff
names(index2) <- gain2$table$trait
GEBV2 <- blup(prep1, geno, what="BV", index.coeff=index2)
```

The following code helps visualize how the ranking of genotypes is
different with the restricted index.

```
# Rank genotypes from high to low 
GEBV1$y <- rank(-GEBV1$value)
GEBV2$y <- rank(-GEBV2$value)

seg1 <- merge(GEBV1[,c("id","y")],GEBV2[,c("id","y")],by="id")
colnames(seg1) <- c("id","y","yend")
plot.data <- data.frame(seg1[seg1$y <= 10 | seg1$yend <= 10,],x=0,xend=1)

library(ggplot2)
ggplot(plot.data,aes(x=x,y=y,xend=xend,yend=yend)) + geom_segment() + theme_bw() + 
  scale_y_reverse(lim=c(20,0),breaks=c(1,5,10,15,20),labels=c(1,5,10,15,20),minor_breaks = NULL,name="Rank",
                  sec.axis = sec_axis(trans=~.*1,breaks=c(1,5,10,15,20),labels=c(1,5,10,15,20))) +
  scale_x_continuous(breaks=c(0,1),labels=c("unrestricted","restricted"),name="") +
  theme(axis.text=element_text(size=13),axis.title=element_text(size=13))
```

Now that we have a GEBV for yield and maturity, we can add fry color
with equal weight to yield in the index. This is accomplished by giving
a list of class “prep” objects (the output from `blup_prep`)
to the `blup` command: the first one is from above for yield
and maturity, and the second one is for fry color. The same genetic
model (i.e., with dominance) is needed for all traits to combine
them.

```
fry1 <- Stage1(filename=pheno.file,traits="fry.color",
              effects=effects)
fry2 <- Stage2(data=fry1$blues, vcov=fry1$vcov, geno=geno, non.add="dom")
fry.prep <- blup_prep(fry1$blues, fry1$vcov, geno, fry2$vars)

index3 <- c(index2, fry.color=as.numeric(index2["total.yield"]))
index3
```

```
##   total.yield vine.maturity     fry.color 
##         0.870        -0.494         0.870
```

```
GEBV3 <- blup(data=list(prep1,fry.color=fry.prep), geno=geno, what="BV",
              index.coeff=index3)
```

### Genomic prediction with secondary traits

The “mask” argument for `blup_prep` makes it easy to
investigate the potential benefit of using a correlated, secondary trait
to improve genomic selection. For example, many plant breeding programs
are exploring the use of spectral measurements from high-throughput
phenotyping platforms to improve selection for yield. The following
example is based on data from Rutkoski et al. (2016), who showed that
canopy temperature (CT) during grain fill was predictive of yield in
wheat. The G matrix and Stage 1 BLUEs from the drought and extreme
drought environments are distributed with the package. As with the
potato dataset in Vignette 1, including the Stage 1 errors in Stage 2
lowers the AIC substantially.

```
data(wheat) #load the wheat data
head(wheat.blues)
```

```
##       env      id trait      BLUE
## 1 drought 6569128    GY  3.395834
## 2 drought 6569128    CT 36.950000
## 3 drought 6688880    GY  3.494048
## 4 drought 6688880    CT 37.902333
## 5 drought 6688916    GY  3.459078
## 6 drought 6688916    CT 38.706000
```

```
ans2a <- Stage2(data=wheat.blues, vcov=wheat.vcov, geno=wheat.geno,
                non.add="none")
ans2b <- Stage2(data=wheat.blues, geno=wheat.geno, non.add="none")

data.frame(vcov=c(TRUE,FALSE), AIC=c(ans2a$aic,ans2b$aic))
```

```
##    vcov       AIC
## 1  TRUE -16.97154
## 2 FALSE 539.30801
```

Because the wheat lines are inbred, the genetic residual option in
StageWise would be appropriate for modeling non-additive values, but
this led to convergence problems with ASReml-R. Thus, non-additive
effects were omitted using the argument non.add=“none”. Genomic
heritability was 0.45-0.50 for yield and canopy temperature, with an
additive genetic correlation of -0.81.

```
summary(ans2a$vars)
```

```
## $var
##                 GY   CT
## env          0.621 1.58
## additive     0.101 0.61
## g x env      0.048 0.44
## Stage1.error 0.055 0.30
## 
## $PVE
##                 GY    CT
## additive     0.495 0.454
## g x env      0.237 0.326
## Stage1.error 0.269 0.221
## 
## $cor.mat
##        GY     CT
## GY  1.000 -0.807
## CT -0.807  1.000
```

Now on to the genomic predictions. First we will do a tenfold cross
validation without using CT data for the selection candidates, which can
be called marker-based selection (MBS, see Vignette 1). Since the goal
is yield prediction, the index coefficients are 1 and 0 for GY and CT,
respectively.

```
id <- unique(wheat.blues$id)
N <- length(id)
folds <- split(sample(id),cut(1:N,10))
MBS <- NULL
for (i in 1:10) {
  prep <- blup_prep(wheat.blues, wheat.vcov, wheat.geno, ans2a$vars, 
                    mask=data.frame(id=folds[[i]]))
  pred <- blup(prep, geno=wheat.geno, what="BV", 
               index.coeff=c(GY=1, CT=0))
  MBS <- rbind(MBS, pred[pred$id %in% folds[[i]],])
}
```

In the above code, the “mask” argument for `blup_prep`
only has the variable “id”, which means that all Stage 1 BLUEs for those
individuals are masked. To only mask grain yield and use CT as a
secondary trait for marker-assisted selection (MAS), a second variable
named “trait” is used.

```
MAS <- NULL
for (i in 1:10) {
  prep <- blup_prep(wheat.blues, wheat.vcov, wheat.geno, ans2a$vars, 
                    mask=data.frame(id=folds[[i]], trait="GY"))
  pred <- blup(prep, geno=wheat.geno, what="BV", 
               index.coeff=c(GY=1, CT=0))
  MAS <- rbind(MAS, pred[pred$id %in% folds[[i]],])
}

ans <- merge(MBS,MAS,by="id")

ggplot(ans,aes(x=r2.x, y=r2.y)) + geom_hex() + coord_fixed(ratio=1) + geom_line(data=data.frame(x=c(0.2,0.8),y=c(0.2,0.8)),mapping=aes(x=x,y=y),linetype=2) +  ggtitle("Reliability") +
  xlab("MBS") + ylab("MAS")
```

The above figure shows that using CT increased the reliability of
genomic prediction, by 0.2 on average.
