# Supplementary material for: Fully efficient, two-stage analysis of multi-environment trials with directional dominance and multi-trait genomic selection
Source: Theor Appl Genet. 2023 Mar 22;136(4):65. doi: 10.1007/s00122-023-04298-x (PMC10033618; doi:10.1007/s00122-023-04298-x)
Supplement: Supplementary file 3 — Supplementary file3 (ZIP 14439 KB) [file 122_2023_4298_MOESM3_ESM.zip › StageWise/Vignette1.html]

Vignette 1: Single trait analysis with homogeneous GxE


# Vignette 1: Single trait analysis with homogeneous GxE

### Preface

This vignette illustrates basic features of the package using a
potato breeding dataset of 943 genotypes (i.e., clones), with phenotype
data from six years at one location. It is an updated version of the
dataset published by Endelman et
al. (2018). This vignette covers single trait analysis, under the
assumption that all environments have the same genetic correlation
(i.e., compound symmetry). The analysis of multiple locations with
different correlations are covered in Vignette
2, and the analysis of correlated traits is covered in Vignette
3.

There are five main functions in the package:

- `read_geno`
- `Stage1`
- `Stage2`
- `blup_prep`
- `blup`

The package depends on ASReml-R (version 4.1.0.148 or later), which
requires a license from VSN
International.

### Stage 1

In Stage 1, the data for each environment are analyzed independently,
which allows for the selection of different models tailored to different
experimental designs and patterns of spatial variation. The
`Stage1` function in the package offers a number of commonly
used analysis methods, but it also possible to use other software for
Stage 1. For a linear model with only fixed and i.i.d. random effects,
the argument `solver="asreml"` triggers the use of ASReml-R
for variance component estimation. Another option is
`solver="spats"`, which triggers the use of R package SpATS
to fit a 2D spline (in addition to fixed or i.i.d. effects). Regardless
of which solver is used, at least some of the individuals should be
replicated (which includes augmented designs with repeated checks). If
you have no replication within environment, skip Stage 1 and go to Stage
2.

There are two required columns in the CSV file of phenotype data used
in `Stage1`: “id” contains the individual identifier and is
matched against the information from the genotype input file; and “env”
is the name of the environment, which is typically a location x year
combination. (This vignette illustrates the analysis of multiple years
from one location, or when multiple locations are similar enough that
the genotype x location effect can be neglected. For the analysis of
correlated locations, consult Vignette
2 after you complete this vignette.) The other columns in the input
file contain traits, cofactors, or covariates.

The phenotypes for this tutorial are based on six years (2015-2020)
of variety trials at the Hancock Research Station of the University of
Wisconsin. Data for the first five years are in the file “pheno1a”,
which includes a column with an incomplete blocking factor. The 2020
data are provided in the file “pheno1b” and consists of two partially
replicated trials (preliminary and advanced), with row and range
information to illustrate spatial analysis.

- total yield (Mg per ha)
- vine maturity (1=early to 9=late)
- fry color, measured in units of Hunter Lightness (L) after 6
  months of storage

The data files also contain the stand count for each plot (out of 15
total plants), which is included as a covariate in the Stage 1
model.

```
pheno1a.file <- system.file("vignette_data", "pheno1a.csv", package = "StageWise")
pheno1a <- read.csv(pheno1a.file)
kable(head(pheno1a))
```

| env | id | block | total.yield | vine.maturity | fry.color | stand.count |
| --- | --- | --- | --- | --- | --- | --- |
| Hancock15 | A00188-3C | 2 | 68.1 | 8 | 47.8 | 15 |
| Hancock15 | A00188-3C | 3 | 71.2 | 8 | 47.3 | 15 |
| Hancock15 | A01143-3C | 2 | 109.2 | 8 | 48.4 | 15 |
| Hancock15 | A01143-3C | 3 | 105.3 | 9 | 51.9 | 15 |
| Hancock15 | A09037-6C | 1 | 70.3 | 5 | 53.9 | 15 |
| Hancock15 | AAF07847-2 | 1 | 77.6 | 8 | 49.0 | 15 |

```
pheno1b.file <- system.file("vignette_data", "pheno1b.csv", package = "StageWise")
pheno1b <- read.csv(pheno1b.file)
kable(head(pheno1b))
```

| env | expt | row | range | id | total.yield | vine.maturity | fry.color | stand.count |
| --- | --- | --- | --- | --- | --- | --- | --- | --- |
| Hancock20 | prelim | 1 | 1 | W17037-24 | 44.7 | 3 | 58.0 | 15 |
| Hancock20 | prelim | 1 | 2 | W17062-11 | 38.8 | 4 | 61.5 | 13 |
| Hancock20 | prelim | 1 | 3 | W17064-7 | 36.8 | 2 | 61.2 | 15 |
| Hancock20 | prelim | 1 | 4 | W17039-15 | 45.4 | 3 | 59.6 | 15 |
| Hancock20 | prelim | 1 | 5 | W17039-30 | 47.1 | 5 | 61.9 | 15 |
| Hancock20 | prelim | 1 | 6 | W17039-7 | 64.8 | 3 | 61.4 | 15 |

```
tmp <- merge(pheno1a[,c("env","id")],pheno1b[,c("env","id")],all=T)
library(ggplot2)
ggplot(data=tmp,aes(x=env)) + geom_bar() + 
  ylab("Number of plots") + xlab("Environment") + theme_bw() + 
  theme(axis.text.x=element_text(angle=90,vjust=0.5,size=10))
```

As is typical of breeding trials, the majority of clones were tested
in one year and then dropped, but there is sufficient replication across
years to estimate genotype x env interactions.

A data frame with variables “name”, “fixed”, and “factor” is used to
specify which columns of the input file should be included as covariates
or cofactors, and whether the effects are fixed or random. We begin with
analysis of the 2015-2019 data, using “block” and “stand.count” as
cofactor and covariate, respectively:

```
effects <- data.frame(name=c("block","stand.count"),
                      fixed=c(FALSE,TRUE),
                      factor=c(TRUE,FALSE))
effects
```

```
##          name fixed factor
## 1       block FALSE   TRUE
## 2 stand.count  TRUE  FALSE
```

```
library(StageWise)
ans1a <- Stage1(filename=pheno1a.file,traits="total.yield",
                effects=effects,solver="asreml")
```

```
## Online License checked out Fri Jan 20 20:15:05 2023
## Online License checked out Fri Jan 20 20:15:05 2023
```

The `Stage1` function returns a list with several results.
List element “blue” is a data frame of the individual BLUEs per
environment. Element “fit” contains the broad-sense heritability on a
plot basis (H2) and the AIC.

```
head(ans1a$blues)
```

```
##         env          id      BLUE
## 1 Hancock15   A00188-3C  66.81187
## 2 Hancock15   A01143-3C 104.41187
## 3 Hancock15   A09037-6C  76.55071
## 4 Hancock15  AAF07847-2  83.85071
## 5 Hancock15  AC01144-1W  70.81187
## 6 Hancock15 Accumulator  83.36187
```

```
ans1a$fit
```

```
##            env   H2   AIC
## 1    Hancock15 0.71 531.6
## 364  Hancock16 0.72 436.7
## 668  Hancock17 0.70 284.0
## 1029 Hancock18 0.83 246.4
## 1368 Hancock19 0.76 340.8
```

To check for outliers and normality of the residuals, use the plots
contained in list element “resid”:

```
ans1a$resid$boxplot
```

```
ans1a$resid$qqplot
```

The reserved word “expt” in the input file, which is short for
"experiment”, directs Stage1 to fit separate models for each experiment
within an environment. Then, in a second step, a single BLUE for each
genotype in that environment is estimated, including the full var-cov
matrix. Compared to simply using “expt” as a factor in a single step, this
two-step procedure within Stage1 allows for separate spatial models.

The 2020 data file contains two experiments: a preliminary and
advanced trial. Here is a comparison of using random row and range
effects vs. a 2D spline, which requires the additional argument
`spline` to indicate the names of the variables in the input
file with the x and y coordinates.

```
effects <- data.frame(name=c("row","range","stand.count"),
                      fixed=c(FALSE,FALSE,TRUE),
                      factor=c(TRUE,TRUE,FALSE))
effects
```

```
##          name fixed factor
## 1         row FALSE   TRUE
## 2       range FALSE   TRUE
## 3 stand.count  TRUE  FALSE
```

```
model1 <- Stage1(filename=pheno1b.file, traits="total.yield",
                effects=effects, solver="asreml")
model1$fit
```

```
##           env     expt   H2   AIC
## 196 Hancock20 advanced 0.86 213.3
## 1   Hancock20   prelim 0.79  45.1
```

```
model2 <- Stage1(filename=pheno1b.file, traits="total.yield",
                effects=effects[3,], solver="spats", spline=c("row","range"))
model2$fit
```

```
##           env     expt   H2
## 196 Hancock20 advanced 0.85
## 1   Hancock20   prelim 0.78
```

```
compare <- merge(model1$blues,model2$blues,by=c("id","env"))
ggplot(data=compare,aes(x=BLUE.x,y=BLUE.y)) + geom_point() + xlab("i.i.d. Random Effects") + ylab("2D Spline") + theme_bw() + geom_abline(intercept=0,slope=1) + coord_cartesian(xlim=c(25,90),ylim=c(25,90)) + ggtitle("2020 Yield BLUEs (Mg/ha)")
```

The above figure shows that the BLUEs are similar with the two
different models. A figure showing the 2D spline and spatial
distribution of residuals is also returned when SpATS is used:

```
model2$resid$spatial$advanced
```

Before proceeding to Stage 2, the BLUEs and variance-covariance
matrices from the 2015-19 analysis and 2020 analysis need to be
combined. (If other software is used for Stage 1, it can be incorporated
in a similar manner.) The model with i.i.d. row and column effects is
selected based on the higher estimate for H2.

```
stage1.blues <- rbind(ans1a$blues,model1$blues)
stage1.vcov <- c(ans1a$vcov,model1$vcov)
```

### Marker data

The `read_geno` function reads bi-allelic marker data as a
CSV file. If you intend to run GWAS, the option `map=TRUE`
indicates the first three columns of the input file are the marker name,
chromosome, and position, followed by columns for the individuals. Map
information is not used for genomic prediction and can be omitted by
using `map=FALSE`, in which case the first column is the
marker name and subsequent columns are individuals. The marker data
should represent allele dosage, with numeric values between 0 and
ploidy. (For compatibility with other software, the coding {-1,0,1} is
also allowed for diploids.)

The potato marker data were generated using an Infinium SNP array.
Most clones were genotyped with Version 3 (V3) of the array, but some
were genotyped with an earlier version (V2). Data from the two different
versions were combined via BLUP using the function
`merge_impute` from R package polyBreedR.

```
geno.file <- system.file("vignette_data", "geno1.csv", package = "StageWise")
geno <- read.csv(geno.file,check.names=F)
geno[1:4,1:6]
```

```
##                marker chrom position AF5392-8 AF5393-1 AF5429-3
## 1 solcap_snp_c2_51460 chr01   449027        0        1     1.03
## 2 solcap_snp_c2_36608 chr01   508800        1        0     2.00
## 3 solcap_snp_c2_36615 chr01   510745        1        0     2.03
## 4 solcap_snp_c2_36658 chr01   527068        4        3     3.00
```

```
geno <- read_geno(filename=geno.file, ploidy=4, map=TRUE, min.minor.allele=5, 
                  dominance=T)
```

```
## Minor allele threshold = 5 genotypes
## Number of markers = 12242
## Number of genotypes = 943
```

The function `read_geno` computes genomic relationship
matrices from the markers. At a minimum, the additive (G) matrix is
computed. When `dominance=TRUE`, the dominance (D) matrix is
also computed. These matrices and other information needed for the
`Stage2` function are stored in the returned object as an S4
class.

The command `inbreeding` returns genomic inbreeding
coefficients, estimated from either the diagonal elements of the G
matrix or the average dominance coefficient. As shown below, the two
methods are very similar and have the same population mean. The negative
inbreeding coefficient indicates excess heterozygosity relative to
panmictic equilibrium, which may be expected when there is inbreeding
depression.

```
x <- inbreeding(geno)
head(x)
```

```
##                  F.G         F.D
## AF5392-8 -0.07650919 -0.07976518
## AF5393-1 -0.10542949 -0.10912896
## AF5429-3 -0.06456956 -0.06159264
## AF5445-2 -0.08590067 -0.08484250
## AF5450-7 -0.09444887 -0.09295317
## AF5484-3 -0.11489437 -0.11669210
```

```
apply(x,2,mean)
```

```
##         F.G         F.D 
## -0.07786684 -0.07786684
```

```
ggplot(x,aes(x=F.G,y=F.D)) + geom_hex()
```

### Stage 2

The `Stage2` function uses the BLUEs from Stage 1 as the
response variable, as well as their variance-covariance matrix to
partition micro-environmental variation from GxE. It is also possible to
run `Stage2` without the covariance of the BLUEs from Stage
1, e.g., when there are no replicated entries within environment. In
this case, use `vcov=NULL`. The benefit of including the
covariance information is reflected in the lower value of AIC, which is
a penalized likelihood to measure goodness-of-fit.

```
ans2a <- Stage2(data=stage1.blues, vcov=NULL)
ans2b <- Stage2(data=stage1.blues, vcov=stage1.vcov)

data.frame(vcov=c(FALSE,TRUE), AIC=c(ans2a$aic,ans2b$aic))
```

```
##    vcov      AIC
## 1 FALSE 9634.154
## 2  TRUE 9535.647
```

```
kable(summary(ans2a$vars))
```

|  | Variance | PVE |
| --- | --- | --- |
| env | 60.5 | NA |
| genotype | 82.0 | 0.557 |
| residual | 65.1 | 0.443 |

```
kable(summary(ans2b$vars))
```

|  | Variance | PVE |
| --- | --- | --- |
| env | 60.0 | NA |
| genotype | 71.3 | 0.500 |
| g x env | 32.6 | 0.228 |
| Stage1.error | 38.8 | 0.272 |

Several other pieces of information are in the list output from
`Stage2`. The variance components are contained in “vars” as
an S4 class, which is used by the `blup_prep` function (see
below). As shown above, the `summary` command returns a
matrix with two columns: the first is the variance, in units of the
trait; the second is the proportion of variance excluding the
environment effect, which makes the result for genotype comparable to
heritability (environment basis). Including the Stage1 var-cov matrix
for the BLUEs enables partitioning of the residual into GxE and Stage1
error.

The above Stage 2 analysis did not include the marker data. To
partition the genotype effects into additive and non-additive effects,
the output from `read_geno` is included in the function call.
StageWise has two ways of modeling non-additive effects. The argument
non.add=“g.resid” leads to a genetic residual, with independent and
identically distributed (iid) effects. When non.add=“dom” (which
requires that `read_geno` was run with dominance=TRUE), the
covariance of the non-additive effects follows the D matrix. To omit
non-additive effects, use non.add=“none”. The AIC can be used to assess
which model is better.

The following example also shows the `silent=FALSE` option
(default is TRUE), which shows the convergence progress from
ASReml-R.

```
ans2c <- Stage2(data=stage1.blues,vcov=stage1.vcov,geno=geno,
                silent=FALSE, non.add="g.resid")
```

```
## Model fitted using the sigma parameterization.
## ASReml 4.1.0 Fri Jan 20 20:15:47 2023
##           LogLik        Sigma2     DF     wall    cpu
##  1     -3748.926           1.0   1209 20:15:51    4.0
##  2     -3647.012           1.0   1209 20:15:54    3.1
##  3     -3544.979           1.0   1209 20:15:57    3.1
##  4     -3492.256           1.0   1209 20:16:00    3.1
##  5     -3477.411           1.0   1209 20:16:04    3.0
##  6     -3476.442           1.0   1209 20:16:07    3.1
##  7     -3476.415           1.0   1209 20:16:10    3.1
##  8     -3476.414           1.0   1209 20:16:13    3.0
```

```
ans2d <- Stage2(data=stage1.blues,vcov=stage1.vcov,geno=geno,
                silent=FALSE, non.add="dom")
```

```
## Model fitted using the sigma parameterization.
## ASReml 4.1.0 Fri Jan 20 20:16:14 2023
##           LogLik        Sigma2     DF     wall    cpu
##  1     -3775.931           1.0   1208 20:16:32   17.9
##  2     -3662.898           1.0   1208 20:16:48   15.9
##  3     -3548.042           1.0   1208 20:17:04   15.9
##  4     -3486.962           1.0   1208 20:17:20   15.6
##  5     -3468.704           1.0   1208 20:17:35   15.6
##  6     -3467.174           1.0   1208 20:17:51   15.6
##  7     -3467.096           1.0   1208 20:18:07   15.5
##  8     -3467.091           1.0   1208 20:18:22   15.6
##  9     -3467.091           1.0   1208 20:18:38   15.5
```

```
data.frame(non.add=c("g.resid","dom"),AIC=c(ans2c$aic,ans2d$aic))
```

```
##   non.add      AIC
## 1 g.resid 6958.828
## 2     dom 6940.182
```

Based on the AIC values, we select the model with dominance. To
compare AIC values for models with marker data to models without marker
data, one needs to ensure that all individuals analyzed in Stage1 are in
the marker data file. Because Stage2 excludes ungenotyped individuals
from the analysis, the populations will not be the same. In this potato
dataset, there are 1294 clones in the phenotype file but only 943 with
marker data.

```
kable(summary(ans2c$vars))
```

|  | Variance | PVE |
| --- | --- | --- |
| env | 51.7 | NA |
| additive | 40.4 | 0.290 |
| g.resid | 33.3 | 0.239 |
| g x env | 32.1 | 0.230 |
| Stage1.error | 33.6 | 0.241 |

```
kable(summary(ans2d$vars))
```

|  | Variance | PVE |
| --- | --- | --- |
| env | 51.0 | NA |
| additive | 49.1 | 0.335 |
| dominance | 17.8 | 0.122 |
| heterosis | 3.8 | 0.026 |
| g x env | 42.0 | 0.287 |
| Stage1.error | 33.6 | 0.230 |

The output for the dominance model includes rows for “dominance” and
“heterosis”; the former is based on the variance of a random effect with
zero mean, while the latter is due to the mean (Varona et
al. 2018).

### Pedigree data

Estimating additive relationships using both marker and pedigree data
is often beneficial because they have complementary properties. Unlike
the G matrix, the A matrix is “sparse”, meaning it has zero covariance
between unrelated individuals. However, the A matrix does not account
for segregation within biparental families or capture LD between
founders. The G and A matrices can be combined into an “H” matrix, which
also allows ungenotyped individuals to be included in the relationship
matrix (Legarra et
al. 2009; Christensen and Lund
2010). (The inclusion of ungenotyped individuals is only available
with the genetic residual model.)

To illustrate this feature, a three-column pedigree file for the
potato population is included with the package. When combining the G and
A matrices, their relative weights must be specified using the argument
`w` in `read_geno`, such that H = (1-w)G + wA.

```
ped.file <- system.file("vignette_data", "ped.csv", package = "StageWise")
ped <- read.csv(ped.file)
geno2 <- read_geno(geno.file,ploidy=4,map=TRUE,ped=ped,w=0.1,dominance = TRUE)
```

```
## Minor allele threshold = 5 genotypes
## Number of markers = 12242
## Number of genotypes = 943
```

```
ans2e <- Stage2(data=stage1.blues,vcov=stage1.vcov,geno=geno2,non.add="dom")
ans2e$aic
```

```
## [1] 6935.89
```

```
kable(summary(ans2e$vars))
```

|  | Variance | PVE |
| --- | --- | --- |
| env | 51.3 | NA |
| additive | 56.9 | 0.385 |
| dominance | 13.8 | 0.093 |
| heterosis | 3.5 | 0.024 |
| g x env | 40.1 | 0.271 |
| Stage1.error | 33.6 | 0.227 |

The above result shows that blending G and A at w=0.1 slightly
reduced the AIC and shifted variance from the non-additive to additive
component. One way to select the blending parameter is based on AIC.
When a vector of w values is provided to `read_geno`, the
function returns a list output corresponding to those values. For
numerical conditioning, a minimum threshold of 1e-5 is used for w.

```
w.vec <- c(1e-5, seq(0.2,0.8,by=0.2))
geno <- read_geno(geno.file,ploidy=4,map=TRUE,ped=ped,w=w.vec,dominance=TRUE)
```

```
## Minor allele threshold = 5 genotypes
## Number of markers = 12242
## Number of genotypes = 943
```

```
result <- data.frame(w=w.vec, aic=numeric(5), h2=numeric(5))
ans2 <- vector("list",5)
for (i in 1:5) {
  ans2[[i]] <- Stage2(data=stage1.blues,vcov=stage1.vcov,geno=geno[[i]],non.add="dom")
  result$aic[i] <- ans2[[i]]$aic
  result$h2[i] <- summary(ans2[[i]]$vars)[2,2]
}
```

```
axis.scaling <- diff(range(result$h2))/diff(range(result$aic))
result$y2 <- (result$aic-min(result$aic))*axis.scaling + min(result$h2)
y2lab <- round(seq(min(result$aic),max(result$aic),length.out=5))
y2axis <- y2lab-min(result$aic) + min(result$h2)/axis.scaling

ggplot(result) + geom_line(mapping=aes(x=w,y=h2)) + geom_line(mapping=aes(x=w,y=y2),colour="red") + scale_y_continuous(name="Genomic h2",sec.axis=sec_axis(trans~./axis.scaling,name="AIC",breaks=y2axis,labels=y2lab)) + theme_bw() +  
  theme(axis.text.y.right=element_text(colour="red"),axis.title.y.right=element_text(colour="red")) + ggtitle("Blending G and A for Yield")
```

Based on the above figure, the blending parameter w=0.4 is chosen, at
which there is no longer much dominance variance. The optimal value for
w will not be the same for all traits.

```
w.vec[3]
```

```
## [1] 0.4
```

```
genoH <- geno[[3]] 
ans2H <- ans2[[3]]
kable(summary(ans2H$vars))
```

|  | Variance | PVE |
| --- | --- | --- |
| env | 51.5 | NA |
| additive | 74.2 | 0.494 |
| dominance | 5.0 | 0.033 |
| heterosis | 2.9 | 0.019 |
| g x env | 34.5 | 0.230 |
| Stage1.error | 33.6 | 0.224 |

To include ungenotyped individuals in the H matrix, put a fourth
column in the pedigree data frame with binary (0/1) values to indicate
which individuals should be included.

### BLUP Reliability

The calculation of BLUPs is split into two functions:
`blup_prep` and `blup`. The computationally
intensive steps occur in `blup_prep`, which combines the
phenotype and genotype information used in `Stage2` with the
variance component estimates to estimate the var-cov matrix of the
predicted random effects. The `blup` command extracts the
appropriate linear combination of predictions based on the argument
“what”, which has 5 possible values:

- AV = additive values
- BV = breeding values
- GV = genotypic values
- AM = additive marker effects
- DM = dominance marker effects

The “values” are properties of individuals, as opposed to markers.
Breeding values should be used for parent selection, while genotypic
values should be used for clone selection. For diploids, the AV and BV
are equivalent. For polyploids, if the dominance model was used, the BV
includes a portion of the dominance. GV is the sum of additive and
non-additive values. When predicting values, the software also returns
the predicted reliability r2, which is the squared correlation between
the true and predicted values (assuming the model is correct).

The following code illustrates the prediction of genotypic
values:

```
prep1 <- blup_prep(data=stage1.blues,
              vcov=stage1.vcov,
              geno=genoH,
              vars=ans2H$vars)
GV1 <- blup(prep1, geno=genoH, what="GV")
kable(head(GV1),digits=2)
```

| id | value | r2 |
| --- | --- | --- |
| AF5392-8 | 54.39 | 0.56 |
| AF5393-1 | 54.95 | 0.70 |
| AF5429-3 | 62.97 | 0.74 |
| AF5445-2 | 72.00 | 0.70 |
| AF5450-7 | 67.85 | 0.70 |
| AF5484-3 | 58.92 | 0.78 |

The figure below illustrates how the reliability of genotypic value
predictions is typically higher when using marker data because it
improves estimation of the additive component.

```
#predict genotypic values without marker data
prep2 <- blup_prep(data=stage1.blues,
              vcov=stage1.vcov,
              vars=ans2b$vars)
GV2 <- blup(prep2, what="GV")

plot.data <- merge(GV2,GV1,by="id")
ggplot(plot.data,aes(x=r2.x,y=r2.y)) + geom_point() + ggtitle("GV Reliability") + theme_bw() + 
  xlab("Without markers") + ylab("With markers") + coord_fixed(ratio=1) + ylim(0.4,1) + xlim(0.4,1) + geom_line(data=data.frame(x=c(0.4,1),y=c(0.4,1)),mapping=aes(x=x,y=y),linetype=2)
```

The `blup_prep` function allows for masking the phenotypes
of some individuals before making the predictions, which allows for
cross-validation. All else being equal, predictions for individuals
without phenotypes, which is called marker-based selection, have lower
reliability than predictions for individuals with phenotypes, which is
called marker-assisted selection. To illustrate, we will mask the
phenotypes for the most recent cohort of breeding lines in the dataset
(which have names beginning with “W17”) and compare with the previous
prediction.

```
id <- stage1.blues$id
mask <- data.frame(id=unique(id[substr(id,1,3)=="W17"]))
head(mask)
```

```
##          id
## 1  W17037-1
## 2 W17037-10
## 3 W17037-11
## 4 W17037-12
## 5 W17037-13
## 6 W17037-15
```

```
prep3 <- blup_prep(data=stage1.blues,
              vcov=stage1.vcov,
              geno=genoH,
              vars=ans2H$vars,
              mask=mask)
GV3 <- blup(prep3, geno=genoH, what="GV")

plot.data <- merge(GV3, GV1, by="id")
plot.data <- plot.data[plot.data$id %in% mask$id,]

ggplot(plot.data,aes(x=r2.x,y=r2.y)) + geom_point() + theme_bw() + ggtitle("Reliability") +
  xlab("MBS") + ylab("MAS") + coord_fixed(ratio=1) + geom_line(data=data.frame(x=c(0.3,0.8),y=c(0.3,0.8)),mapping=aes(x=x,y=y),linetype=2)
```

Using the `mask` argument, one can also specify that
individuals are masked only in some environments, which is useful for
assessing the accuracy of prediction into new environments based on
phenotypes in other environments. This idea is revisited in Vignette
2.

### Marker effects and GWAS

Using what=“AM” or “DM” leads to the prediction of additive or
dominance marker effects, respectively, in `blup`. This can
be a convenient way to save the results of a training set analysis to
predict future individuals. Multiplying the additive marker effects by
the matrix of (centered) marker dosages to obtain additive values is
equivalent (up to a constant) to directly predicting the additive values
using what=“AV”, provided there has been no blending with the pedigree
relationship matrix:

```
#w = 0
prep <- blup_prep(data=stage1.blues,vcov=stage1.vcov,geno=geno[[1]],vars=ans2[[1]]$vars)
marker.effects <- blup(data=prep, geno=geno[[1]], what="AM")
head(marker.effects)
```

```
##                marker chrom position       effect
## 1 solcap_snp_c2_51460 chr01   449027 -0.008699366
## 2 solcap_snp_c2_36608 chr01   508800  0.023545090
## 3 solcap_snp_c2_36615 chr01   510745  0.003188108
## 4 solcap_snp_c2_36658 chr01   527068  0.044723545
## 5 solcap_snp_c1_10930 chr01   566972  0.023206061
## 6       PotVar0120126 chr01   603013  0.001992442
```

```
AV1 <- predict(geno[[1]], marker.effects)

#compare with G-BLUP
AV2 <- blup(data=prep, geno=geno[[1]], what="AV")
plot.data <- merge(AV1, AV2, by="id")
ggplot(plot.data,aes(x=value.x,y=value.y)) + geom_point() + xlab("RR-BLUP") + ylab("G-BLUP") + theme_bw()
```

This equivalency also holds for genotypic values (A + D):

```
DM <- blup(data=prep, geno=geno[[1]], what="DM")
DV1 <- predict(geno[[1]], DM)
GEGV1 <- data.frame(id=AV1$id, value=AV1$value + DV1$value)

GEGV2 <- blup(data=prep, geno=geno[[1]], what="GV")
plot.data <- merge(GEGV1,GEGV2,by="id")

ggplot(plot.data,aes(x=value.x,y=value.y)) + geom_point() + xlab("RR-BLUP") + ylab("GD-BLUP") + theme_bw()
```

The marker effects can be standardized to compute GWAS -log10(p)
scores that are equivalent to the traditional fixed effect method (Duarte et
al. (2014); Bernal Rubio
et al. 2016). This calculation is easily parallelized, and the
argument `gwas.ncore` specifies how many cores to use (the
default is 0, which skips computing the GWAS scores). The
`gwas_threshold` command computes the -log10(p) threshold for
QTL discovery based on an effective number of markers (Moskvina and Schmidt,
2008), and `manhattan_plot` displays the result. For
yield there were no significant QTL, so results for the vine maturity
trait are shown instead.

```
effects <- data.frame(name="block",fixed=FALSE,factor=TRUE)
ans1vm <- Stage1(filename=pheno1a.file,traits="vine.maturity",
                effects=effects,solver="asreml")
ans2vm <- Stage2(data=ans1vm$blues, vcov=ans1vm$vcov, geno=geno[[1]], 
                 non.add="dom")

prep <- blup_prep(ans1vm$blues, ans1vm$vcov, geno[[1]], ans2vm$vars)
gwas.ans <- blup(prep, geno[[1]], what="AM", gwas.ncore=2)
head(gwas.ans)
```

```
##                marker chrom position        effect     score
## 1 solcap_snp_c2_51460 chr01   449027 -0.0009276409 0.2699442
## 2 solcap_snp_c2_36608 chr01   508800  0.0030442861 0.7265456
## 3 solcap_snp_c2_36615 chr01   510745  0.0010404348 0.3693139
## 4 solcap_snp_c2_36658 chr01   527068  0.0022755631 0.5325282
## 5 solcap_snp_c1_10930 chr01   566972  0.0013878757 0.2930189
## 6       PotVar0120126 chr01   603013  0.0023887931 0.5896115
```

```
gwas_threshold(geno[[1]], alpha=0.05, n.core=2)
```

```
## [1] 5.103417
```

```
manhattan_plot(gwas.ans, thresh=5.1, rotate.label=TRUE)
```

The GWAS peak on chr05 is near the gene *CDF1*, which is known
to have a large effect on potato maturity (Kloosterman et
al. 2013). The following code extracts the most significant marker
for the large QTL on chr05 and passes it to `Stage2` as a
fixed effect.

```
k <- which.max(gwas.ans$score)
gwas.ans[k,]
```

```
##                   marker chrom position      effect    score
## 5334 solcap_snp_c2_22964 chr05  4422417 -0.01642856 17.05288
```

```
ans2vm.1 <- Stage2(data=ans1vm$blues,
               vcov=ans1vm$vcov,
               geno=geno[[1]],
               fix.eff.marker="solcap_snp_c2_22964",
               non.add="g.resid")

# Fixed effect for the marker
ans2vm.1$fixed$marker
```

```
##                marker     effect
## 1 solcap_snp_c2_22964 -0.7764807
```

```
# Proportion of variance
kable(summary(ans2vm.1$vars))
```

|  | Variance | PVE |
| --- | --- | --- |
| env | 0.485 | NA |
| fixed.marker | 0.313 | 0.123 |
| additive | 0.596 | 0.235 |
| g.resid | 0.445 | 0.176 |
| g x env | 0.297 | 0.117 |
| Stage1.error | 0.884 | 0.349 |

The fixed effect estimate of -0.78 for solcap\_snp\_c2\_22964 implies
that, on average, each additional copy of the alternate allele reduced
vine maturity by 0.78 (on a 1-9 visual scale). According to the
proportion of variance table, the marker accounted for 0.123/(0.123 +
0.235) = 34% of the breeding value.
