# Supplementary material for: Fully efficient, two-stage analysis of multi-environment trials with directional dominance and multi-trait genomic selection
Source: Theor Appl Genet. 2023 Mar 22;136(4):65. doi: 10.1007/s00122-023-04298-x (PMC10033618; doi:10.1007/s00122-023-04298-x)
Supplement: Supplementary file 3 — Supplementary file3 (ZIP 14439 KB) [file 122_2023_4298_MOESM3_ESM.zip › StageWise/StageWise_0.95.tar.gz!/StageWise/docs/manual.pdf]

Package ‘StageWise’

November 27, 2022

Title Two-stage analysis of multi-environment trials for genomic selection and GWAS

Version 0.92

Author Jeffrey B. Endelman

Maintainer Jeffrey Endelman <endelman@wisc.edu>

Description Fully efficient, two stage analysis of multi-
environment trials, including directional dominance and multi-trait genomic selection

Depends R (>= 4.0)

License GPL-3

RoxygenNote 7.1.1

Encoding UTF-8

Imports Matrix, ggplot2, methods, ggrepel, rlang, ggpubr, SpATS, spam, AGHmatrix, MASS, CVXR, ggforce

Suggests knitr, rmarkdown, asreml

Collate 'Stage1.R'
'Stage2.R'
'blup.R'
'blup_prep.R'
'class_geno.R'
'class_genoD.R'
'class_prep.R'
'class_var.R'
'corr.R'
'gain.R'
'gwas_threshold.R'
'inbreeding.R'
'manhattan_plot.R'
'predict.geno.R'
'private_functions.R'
'quantile.geno.R'
'read_geno.R'
'summary.var.R'
'uniplot.R'
'wheat-data.R'

R topics documented:

blup 2

blup_prep . . . 3
class_geno-class . . . 4
class_genoD-class . . . 4
class_prep-class . . . 5
class_var-class . . . 5
corr . . . 6
gain . . . 6
gwas_threshold . . . 7
inbreeding . . . 8
manhattan_plot . . . 8
predict . . . 9
quantile . . . 9
read_geno . . . 10
Stage1 . . . 11
Stage2 . . . 12
summary.var . . . 14
uniplot . . . 14
wheat.data . . . 15

Index **16**

`blup` *BLUP*

Description

BLUP

Usage

```
blup(data, geno = NULL, what, index.coeff = NULL, gwas.ncore = 0L)
```

Arguments

`data`	one object, or list of objects, of `class_prep` from `blup_prep`
`geno`	object of `class_geno` from `read_geno`
`what`	One of the following: AV, BV, GV, AM, DM. See Details.
`index.coeff`	named vector of index coefficients for the locations or traits
`gwas.ncore`	Integer indicating number of cores to use for GWAS (default is 0 for no GWAS).

Details

The argument `what` takes 5 possible values: "AV" (additive value), "BV" (breeding value), "GV" (genotypic value), "AM" (additive marker effect), and "DM" (dominance marker effect). "Values" refer to predictions for individuals, as opposed to markers. Predicted values include the average fixed effect of the environments, whereas predicted marker effects do not. Argument `index.coeff` is a named vector (matching the names of the locations or traits), and the values are interpreted for standardized traits.

When multiple objects of `class_prep` are used for `data`, they must be based on the same marker data and genetic model. Also, reliabilities are not computed.

Value

Data frame of BLUPs

`blup_prep` *Prepare data for BLUP*

Description

Prepare data for BLUP

Usage

```
blup_prep(data, vcov = NULL, geno = NULL, vars, mask = NULL, method = NULL)
```

Arguments

data	data frame of BLUEs from Stage 1
vcov	list of variance-covariance matrices for the BLUEs
geno	object of class_geno from read_geno
vars	object of class_var from Stage2
mask	(optional) data frame with possible columns "id","env","trait"
method	(optional) "MME", "Vinv", NULL (defaut). see Details

Details

The `method` argument can be used to control how the linear system is solved. "MME" leads to inversion of the MME coefficient matrix, while "Vinv" leads to inversion of the overall var-cov matrix for the response vector. If NULL, the software uses whichever method involves inverting the smaller matrix. If the number of random effects (m) is less than the number of BLUEs (n), "MME" is used.

For the multi-location model, if all of the environments for a location are masked, the average of the other locations is used when computed average fixed effects.

Value

Object of class_prep

class_geno-class — *S4 class for marker genotype data*

Description

S4 class for marker genotype data

Slots

- `ploidy` ploidy
- `map` Marker map positions
- `coeff` Coefficients of the marker effects (dim: indiv x marker)
- `scale` Scaling factor between markers and indiv
- `G` Additive relationship matrix (from markers and potentially also pedigree)
- `eigen.G` list of eigenvalues and eigenvectors

class_genoD-class — *S4 class for marker genotype data with dominance*

Description

S4 class for marker genotype data with dominance

Slots

- `ploidy` ploidy
- `map` Marker map positions
- `coeff` Coefficients of the additive marker effects (dim: indiv x marker)
- `scale` Scaling factor between markers and indiv for additive effects
- `G` Additive relationship matrix (from markers and potentially also pedigree)
- `eigen.G` list of eigenvalues and eigenvectors for G
- `coeff.D` coefficients of the dominance marker effects (dim: indiv x marker)
- `scale.D` Scaling factor between markers and indiv for dominance effects
- `D` Dominance relationship matrix
- `eigen.D` list of eigenvalues and eigenvectors for D
- `Fg` genomic inbreeding coefficient (based on dominance)

class_prep-class — *S4 class to prepare for blup*

Description

S4 class to prepare for blup

Slots

- `id` genotype identifiers
- `ploidy` ploidy
- `var.u` variance of random effects
- `var.uhat` variance of BLUPs
- `avg.env` average fixed effect of the environments
- `heterosis` regression coefficients for inbreeding
- `fixed.marker` fixed marker effects
- `B` var-cov matrix for fixed effects
- `random` random effect estimates
- `geno1.var` first var-cov matrix from `class_var`
- `geno2.var` second var-cov matrix from `class_var`
- `model` model from `class_var`

class_var-class — *S4 class for variances*

Description

S4 class for variances

Slots

- `geno1` first genetic effect
- `geno2` second genetic effect
- `model` 0=no markers, 1=add, 2=add+g.resid, 3=add+dom
- `resid` residual
- `diagG` average diagonal element of the G matrix
- `diagD` average diagonal element of the D matrix
- `vars` variances for reporting
- `B` var-cov matrix of fixed effects for gain
- `fix.eff.marker` names of fixed effect markers

corr *Trait correlations*

Description

Trait correlations

Usage

```
corr(vars, traits = NULL, effect = NULL)
```

Arguments

`vars`	object of `class_var` from `Stage2`
`traits`	pair of traits
`effect`	name of effect

Details

Use either the argument `traits` or `effect`, not both. Using `traits` leads to a partitioning of the total correlation between those two traits, based on path analysis, assuming no correlation between the effects of the Stage 2 model. Using `effect` displays the correlation between all traits for that effect. Use the `summary` command to see the names of the possible effects.

Value

matrix

gain *Genetic gain*

Description

Genetic gain for breeding values

Usage

```
gain(input, traits = NULL, coeff = NULL, restricted = NULL, solver = "ECOS")
```

Arguments

`input`	either object of `class_prep` or quad.mat returned by this function
`traits`	optional, plots ellipse tradeoff
`coeff`	optional, index coefficients expressed in genetic standard deviation units
`restricted`	data frame of restricted traits see Details
`solver`	name of convex solver (default is "ECOS")

Details

Optional argument `restricted` is a data frame with columns "trait" and "sign", where the options for sign are "=",">","<", representing equal to zero, non-negative, and non-positive.

Value

List containing

quad.mat quadratic matrix for the ellipsoid

plot ellipse plot

table data frame with gain and coefficients for the traits

`gwas_threshold` *Compute GWAS discovery threshold*

Description

Compute GWAS discovery threshold

Usage

```
gwas_threshold(geno, alpha = 0.05, exclude.chrom = NULL, n.core = 1)
```

Arguments

`geno`	object of `class_geno`
`alpha`	genome-wide significance level
`exclude.chrom`	chromosomes to exclude
`n.core`	number of cores to use

Details

Uses a Bonferroni-type correction based on an effective number of markers that accounts for LD (Moskvina and Schmidt, 2008).

Value

-log10(p) threshold

References

Moskvina V, Schmidt KM (2008) On multiple-testing correction in genome-wide association studies. Genetic Epidemiology 32:567-573. doi:10.1002/gepi.20331

`inbreeding` *Genomic inbreeding coefficient*

Description

Genomic inbreeding coefficient

Usage

```
inbreeding(geno)
```

Arguments

`geno`	object of `class_geno`

Details

Under the additive model, the inbreeding coefficient comes from the diagonal elements of the G matrix according to F = (G-1)/(ploidy-1). For dominance, the inbreeding coefficient is the scaled row-sum of the dominance coefficient matrix.

Value

data frame with F[G] and (when dominance is present) F[D]

`manhattan_plot` *Create Manhattan plot*

Description

Create Manhattan plot

Usage

```
manhattan_plot(data, chrom = NULL, thresh = NULL, rotate.label = FALSE)
```

Arguments

`data`	data frame with columns for marker, chrom, position, and score
`chrom`	optional, to plot only one chromosome
`thresh`	optional, to include horizontal line at discovery threshold
`rotate.label`	TRUE/FALSE whether to rotate x-axis labels to be perpendicular

Details

Assumes position in bp

Value

ggplot2 object

predict *Predict individual values from marker effects*

Description

Predict individual values from marker effects

Arguments

object	object of class_geno
marker.effects	data frame with columns "marker" and "effect"

Details

Use the blup function with what="AM" or "DM" to generate the data frame for marker.effects.

Value

data frame with columns "id" and "value"

quantile *G matrix quantile*

Description

G matrix quantile

Arguments

x	object of class_geno
prob	probability

Details

Unlike the S3 method, prob must have length = 1

Value

data frame with the quantile of the G matrix coefficients for each id

`read_geno` *Read marker genotype data*

Description

Read marker genotype data

Usage

```
read_geno(
  filename,
  ploidy,
  map,
  min.minor.allele = 5,
  w = 1e-05,
  ped = NULL,
  dominance = FALSE
)
```

Arguments

`filename`	Name of CSV file with marker allele dosage
`ploidy`	2,4,6,etc. (even numbers)
`map`	TRUE/FALSE
`min.minor.allele`	threshold for marker filtering (see Details)
`w`	blending parameter (see Details)
`ped`	optional, pedigree data frame with 3 or 4 columns (see Details)
`dominance`	TRUE/FALSE whether to include dominance covariance (see Details)

Details

When `map=TRUE`, first three columns of the file are marker, chrom, position. When `map=FALSE`, the first column is marker. Subsequent columns contain the allele dosage for individuals/clones, coded 0,1,2,...ploidy (fractional values are allowed). The input file for diploids can also be coded using -1,0,1 (fractional values allowed). Additive coefficients are computed by subtracting the population mean from each marker, and the additive (genomic) relationship matrix is computed as G = tcrossprod(coeff)/scale. The scale parameter ensures the mean of the diagonal elements of G equals 1 under panmictic equilibrium. Missing genotype data is replaced with the population mean.

G can be blended with the pedigree relationship matrix (A) by providing a pedigree data frame in ped and blending parameter `w`. The blended relationship matrix is H = (1-w)G + wA. The first three columns of `ped` are id, parent1, parent2. Missing parents must be coded NA. An optional fourth column in binary (0/1) format can be used to indicate which ungenotyped individuals should be included in the H matrix, but this option cannot be combined with dominance. If there is no fourth column, only genotyped individuals are included. If a vector of w values is provided, the function returns a list of `class_geno` objects.

If the A matrix is not used, then G is blended with the identity matrix (times the mean diagonal of G) to improve numerical conditioning for matrix inversion. The default for w is 1e-5, which is somewhat arbitrary and based on tests with the vignette dataset.

When dominance=FALSE, non-additive effects are captured using a residual genetic effect, with zero covariance. If dominance=TRUE, a (digenic) dominance covariance matrix is used instead.

The argument min.minor.allele specifies the minimum number of individuals that must contain the minor allele. Markers that do not meet this threshold are discarded.

Value

Variable of class class_geno.

Stage1 *Stage 1 analysis of multi-environment trials*

Description

Computes genotype BLUEs for each experiment

Usage

```
Stage1(
  filename,
  traits,
  effects = NULL,
  solver = "asreml",
  spline = NULL,
  silent = TRUE,
  workspace = c("500mb", "500mb")
)
```

Arguments

filename	Name of CSV file
traits	trait names (see Details)
effects	data frame specifying other effects in the model (see Details)
solver	one of the following: "asreml","spats"
spline	vector of variable names for 2D spline with SpATS
silent	TRUE/FALSE, whether to suppress REML output
workspace	memory limits for ASRreml-R

Details

The input file must have one column labeled "id" for the individuals and one labeled "env" for the environments. The data for each environment are analyzed independently with a linear mixed model. Although not used in Stage1, to include a genotype x location effect in Stage2, a column labeled "loc" should be present in the input file.

Argument effects is used to specify other i.i.d. effects besides genotype and has three columns: name, fixed, factor. The "name" column is a string that must match a column in the input file. The fixed column is a logical variable to indicate whether the effect is fixed (TRUE) or random (FALSE). The factor column is a logical variable to indicate whether the effect is a factor (TRUE) or numeric (FALSE).

Argument `solver` specifies which software to use for REML. Current options are "asreml" and "spats". For "spats", the argument `spline` must be a vector of length two, with the names of the x and y variables (respectively) for the 2D spline.

The heritability and residuals in the output are based on a random effects model for id.

Missing response values are omitted for single-trait analysis but retained for multi-trait analysis (unless both traits are missing), to allow for prediction in Stage 2.

Argument `workspace` is a vector of length two containing the workspace and pworkspace limits for ASReml-R, with default values of 500mb. If you get an error about insufficient memory, try increasing the appropriate value (workspace for variance estimation and pworkspace for BLUE computation).

For multiple traits, only "asreml" is supported, and only the BLUE model is run, so the returned object does not contain H2.

If the input file has a column "expt", this indicates multiple experiments within environment, which may be needed when using spatial analyses. Each experiment is first analyzed separately, and then the BLUEs from all experiments in one env are jointly analyzed to compute a single BLUE per env. The estimation errors from each experiment are propagated into the multi-expt model.

Value

List containing

blues data frame of BLUEs

vcov list of variance-covariance matrices for the BLUEs, one per experiment (env)

fit data frame with broad-sense H2 (plot basis) and/or AIC

resid For single trait, list of diagnostic plots and data frame of residuals. For multi-trait, list of resid var-cov matrices.

`Stage2` *Stage 2 analysis of multi-environment trials*

Description

Stage 2 analysis of multi-environment trials

Usage

```
Stage2(
  data,
  vcov = NULL,
  geno = NULL,
  fix.eff.marker = NULL,
  silent = TRUE,
  workspace = "500mb",
  non.add = "g.resid",
  max.iter = 20
)
```

Arguments

`data`	data frame of BLUEs from Stage 1 (see Details)
`vcov`	named list of variance-covariance matrices for the BLUEs
`geno`	output from `read_geno`
`fix.eff.marker`	markers in geno to include as additive fixed effect covariates
`silent`	TRUE/FALSE, whether to suppress ASReml-R output
`workspace`	Memory limit for ASRreml-R variance estimation
`non.add`	one of the following: "none","g.resid","dom"
`max.iter`	maximum number of iterations for asreml

Details

Stage 2 of the two-stage approach described by Damesa et al. 2017, using ASReml-R for variance component estimation. The variable `data` has three mandatory column: id, env, BLUE. Optionally, `data` can have a column labeled "loc", which changes the main effect for genotype into a separable genotype-within-location effect, using a FA2 covariance model for the locations. Optionally, `data` can have a column labeled "trait", which uses an unstructured covariance model. The multi-location and multi-trait analyses cannot be combined. Missing data are allowed in the multi-trait but not the single-trait analysis. The argument `geno` is used to partition genetic values into additive and non-additive components. Any individuals in `data` that are not present in `geno` are discarded.

The argument `vcov` is used to partition the macro- and micro-environmental variation, which are called GxE and residual in the output. `vcov` is a named list of variance-covariance matrices for the BLUEs within each environment, with id for rownames (single trait) or id:trait. The order in `vcov` and `data` should match. Both `data` and `vcov` can be created using the function `Stage1`.

Because ASReml-R can only use relationship matrices defined in the global environment, this function creates and then removes global variables when either `vcov` or `geno` is used. By default, the workspace memory for ASReml-R is set at 500mb. If you get an error about insufficient memory, try increasing it. ASReml-R version 4.1.0.148 or later is required.

Value

List containing

aic AIC

vars variance components for `blup_prep`, as variable of class `class_var`

fixed Fixed effect estimates for env and markers

random Random effect predictions

loadings scaled loadings for the FA2 multi-loc model

References

Damesa et al. 2017. Agronomy Journal 109: 845-857. doi:10.2134/agronj2016.07.0395

summary.var *Displays variances and correlations*

Description

Displays variances and correlations

Arguments

`object`	object of `class_var`
`digits`	number of digits for rounding

Details

For a single trait, the 'var' output is a data frame with two columns of information for the various effects: the first is the variance and the second is the proportion of variance explained (PVE), excluding the environment effect. For multiple locations or traits, the 'cor' output is the correlation matrix for additive effects (does not include fixed effect markers). For multiple traits, the variance and PVE results are returned as separate data frames.

Value

List output that varies depending on the situation (see Details)

uniplot *Uniplot for multi-location models*

Description

Displays scaled loadings of the FA2 model

Usage

```
uniplot(loadings, nudge = 0.1)
```

Arguments

`loadings`	scaled factor loadings, from `Stage2`.
`nudge`	distance to nudge labels

Details

The squared radius for each point is the proportion of genetic variance explained by the latent factors. For points on the unit circle, the cosine of the subtended angle equals the correlation.

Value

ggplot2 object

wheat.data *Genomic prediction from secondary traits in wheat*

Description

Canopy temperature (CT) measurements collected during grain fill; used for genomic prediction of grain yield (GY) in wheat. Data come from the drought and extreme drought environments of Rutkoski et al. (2016). The CT phenotype was dated 3/7/2014. Stage 1 BLUEs were computed using rep(trial) as a random effect.

Usage

```
data(wheat)
```

Format

wheat.geno is object of `class_geno`. wheat.blues and wheat.vcov are output from `Stage1`.

References

Rutkoski et al. (2016) G3 (Bethesa) 6:2799–2808. https://doi.org/10.1534/g3.116.032888

Index

∗ **datasets**
wheat.data, 15

blup, 2, *9*
blup_prep, *2*, 3, *13*

class_geno, *2, 3*, *7–11*, *15*
class_geno (class_geno-class), 4
class_geno-class, 4
class_genoD (class_genoD-class), 4
class_genoD-class, 4
class_prep, *2, 3*, *6*
class_prep (class_prep-class), 5
class_prep-class, 5
class_var, *3*, *5, 6*, *13, 14*
class_var (class_var-class), 5
class_var-class, 5
corr, 6

gain, 6
gwas_threshold, 7

inbreeding, 8

manhattan_plot, 8

predict, 9

quantile, 9

read_geno, *2, 3*, 10, *13*

Stage1, 11, *13*, *15*
Stage2, *3*, *6*, *11*, 12, *14*
summary.var, 14

uniplot, 14

wheat.data, 15
